# Synchronous and metachronous gastric gist with pancreatic adenocarcinoma: report of 2 cases and a review of literature 

**Published:** 2015

**Authors:** Marco Fiore, Giorgio de Stefano, Nicola Coppola, Antonio Giorgio

**Affiliations:** 1 *Infectious Disease Unit, University Hospital of Trieste, Italy Piazzale Ospitale 1, 34100, Trieste, Italy*; 2 *Ultrasound Unit for Infectious Diseases, AORN dei Colli, Cotugno Hospital, Via G. Quagliarello 54, 80131 Naples, Italy*; 3 *Department of Mental Health and Public Medicine, Section of Infectious Diseases, Second University of Naples, Via L. Armanni 5, Naples, 80133, Italy.*; 4 *Interventional Ultrasound Unit, Tortorella Clinical Institute, ISSMES Consortium, Via Nicola Aversano 1, 84124 Salerno , Italy*

**Keywords:** GIST, Pancreatic adenocarcinoma, Synchronous cancer, Metachronus cancer

## Abstract

We report two cases of a Gastrointestinal Stromal Tumor (GIST) synchronous and metachronous, respectively, with pancreatic adenocarcinoma. To our knowledge, this is the first report of a GIST involved 3 years after a ductal pancreatic adenocarcinoma. Data from the literature and our cases seem to suggest that incidental GIST may occur synchronously and metachronously with other cancers more frequently than expected. Thus, the patients with a diagnosis of pancreatic adenocarcinoma may have undergone a strict follow up for GIST

## Introduction

 Gastrointestinal Stromal Tumor (GIST) is a rare neoplasm that represents about 0.1%-1.0% of all malignant neoplasms of the gastrointestinal tract (1). Stomach and small bowel are the most frequently affected anatomic sites (2). The literature data reported a synchronism or metachronism between GISTs and other primary cancers. However, there are few data on a possible correlation between GIST and pancreatic cancer. 

In this paper, we describe two patients with a synchronous and metachronous GIST, respectively, with pancreatic adenocarcinoma observed in two Liver Units in Naples. 

## Case Report

**Case 1**

In August 2010, a 63-year-old Caucasian male was admitted with a jaundice of four weeks duration and abdominal pain that lasts a week. Physical examination revealed jaundice and tenderness abdomen. The laboratory data showed a total bilirubin of 11.2 mg/dl (direct bilirubin 5.8 mg/dl), alkaline phosphatase 197 IU/l, aspartate aminotransferase (AST) 111 IU/ml and alanine aminotransferase (ALT) 183 IU/ml (Table 1). An abdominal UltraSonography (US) showed a mild dilation of the biliary tract the VIII segment, a hypoechoic *oval-*shaped mass (short axis diameter of 31 mm and long axis diameter of 39 mm) in the head of Pancreas and an iso-hypoechoic gastric mass of 5 cm. A Contrast Enhanced Ultrasound (CEUS) was performed: the Pancreatic Mass showed in arterial phase an enhancement with fine spot composing a wire aspect and a persisting homogeneously hypoechoic in the late phases suggesting a malignant tumor. The CEUS of the gastric mass showed a rapid arterial enhancement and a slow wash-out. Subsequently an Endo UltraSonography (EUS) with a FNA was performed on the gastric neoplasm with a diagnosis of GIST (T2N0) and on the pancreatic head with a diagnosis of adenocarcinoma (T2N0). A total body MDCT was performed and no other or secondary lesions nor vascular invasions nor linphoadenopathy were observed. Thus, the patient was referred to a tertiary Center for Hepatobiliary and Pancreatic Diseases in the North of Italy, and surgically treated (sub-total gastrectomy and cefalo-pancreatectomy). From that time the patient is followed up and is still alive.

**Table 1.** Demographic, biochemical and ultrasonographic characteristics of the two patients at the time of the diagnosis of pancreatic adenocarcinoma

	**Case n° 1**	**Case n° 2**
Age, year	63	66
Gender	Male	Male
Time of observation	2010	2007
Months between diagnosis of pancreatic adenocarcinoma and GIST	0	39
Red Blood cells, cells/ml	4.45E6	4.30E6
Haemoglobin, g/dl	14.7	14.3
White Blood Cells, cells/ml	4.6E3	4.2E3
Platelet count, cells/ml	242E3	69E3
AST, IU/ml	111 (n.v. 10-40)	35 (n.v. <45)
ALT, IU/ml	183 (n.v. 10-60)	49 (n.v. <63)
Total bilirubin, mg/dl	11,2 mg/dl (n.v <1.2)	1.13 mg/dl (n.v <1)
Direct bilirubin, mg/dl	5.8 mg/dl (n.v. <0,4)	0.55 mg/dl (n.v. <0,2)
ALP, IU/ml	197 (n.v. 38-126)	89 (n.v. 30-122)
γ-GT, IU/ml	406 (n.v. <50)	31 (n.v. <50)
INR (normal value)	1 (0.8-1.3)	1.18 (0.8-1.2)
Ca19-9, IU/ml	647 (n.v. <33)	287.3 (n.v. <37)
HCV Ab	Negative	Positive
Pancreas Ultrasound characteristics	Hypoechoic mass of 31 × 39 mm in the head of pancreas	A hypoechoic, irregular-shaped mass of 4 cm in the body of the Pancreas
CEUS characteristics	Arterial phase and enhancement with fine spot composing a wire aspect, persisting homogeneously hypoechoic in the late phases	A rapid enhancement in arterial phase with a persisting homogeneously hypoechogenecity in the late phases

Footnotes: AST: aspartate transaminase; n.v.: normal value; ALT: alanine transaminase; ALP: Alkaline phosphatase; CA 19-9: Carbohydrate Antigen 19.9; CEUS: Contrast Enhanced Ultrasound; γ-GT: gamma glutamyl transferases; GIST: gastrointestinal stromal tumor; HCV Ab: hepatitis C virus antibodies; INR: International Normalized Ratio

**Case 2**

In March 2007, a 62-year-old Caucasian male followed for a compensated HCV-correlated liver cirrhosis at the Liver Unit of the Second University of Naples. Due to dyspeptic symptoms and weight loss in the last two months, he was evaluated. The patient with a clinical history of diabetes type 2, had a lung adenocarcinoma in 1994 treated with a right upper lobectomy. At the time of our observation, an abdominal US showed a hypoechoic, irregular-shaped mass of 4 cm in the body of the Pancreas. The laboratory data showed an abnormal serum value of CA 19.9 and a leuko- thrombocytopenia (Table 1). A CEUS showed that the pancreatic lesion was hypovascular in the arterial phase with a persisting homogeneously and hypoechogenecity in the late phases suggestive of a malignant tumor. The CT scan confirmed the hypovascularity. The fine needle aspiration biopsy (FNAB) was performed with a diagnosis of ductal pancreatic adenocarcinoma. Subsequently a Radio Frequency Ablation was performed and followed by radiotherapy with systemic and local chemotherapy (infusion of 5-fluorouracil, leucovorin, epirubicin and carboplatin - FLEC regimen).

After three years, in June 2010, a routinely abdominal US showed a mass of 2 cm in celiac trunk area. The CEUS showed a mass with hypo-enhancement in the arterial phase, confirmed by CT scan. After this diagnostic test, the patient underwent laparotomy surgery. An intra-operative biopsy was negative for cancer, but an intra-operative ultrasonography showed a gastric mass of 1.5 cm. During laparotomy, multiple bioptic samples were obtained with the evidence of fibrosis in the celiac area and gastric GIST. After laparotomy, the patient showed decompensation of liver cirrhosis with ascites. Due to decompensation, never resolved, the patient was not able to undergo a new surgical treatment. In April 2011 the patient died due to GIST complications.

## Discussion

In this paper, we described the history of two patients showing a synchronous and metachronous GIST, respectively, with pancreatic adenocarcinoma. It was considered as synchronous every cancer diagnosed at the same time with another malignance. It was considered metachronous every abnormal growth of tissue that followed a previous neoplasm, but was not metastases of the latter; the second malignancy may have the same or different histological type and can occur in the same or different organs as the previous neoplasm but in all cases arises from an independent oncogenic event (3). In the first case of this report (Synchronous cancer), the patient had no history of cancer or liver disease and presented to us with symptoms/signs of a cancer of the pancreatic head, such jaundice; at this time he had also an incidental diagnosis of gastric GIST. In the second case (Metachronous cancer), a diagnosis of pancreatic adenocarcinoma was made in March 2007, in the patient with dyspeptic symptoms and weight loss who also had a history of lung cancer, liver cirrhosis and type 2 diabetes; three years after, in June 2010, in a follow-up screening we find incidentally an asymptomatic gastric GIST. To our knowledge, this is the first case report of a GIST involved 3 years after a ductal pancreatic adenocarcinoma. In fact, in literature there were a few series on synchronous/metachronous GIST and other primary cancers (1, 4, 5, 6-8). Agaimy et co-workers in a case series study of 18 patients, having GIST and other cancer types, found two pancreatic ductal carcinoma associated with gastric GIST, as our two case reports, one simultaneous and the other methacronous (after 10 months of the diagnosis of GIST) (4). Liszka et al. described three simultaneous pancreatic adenocarcinomas that were associated with GIST, and located in the small intestine (5). Other authors described the coexistence of GIST and other cancers, but no one had a pancretic adenocarcinoma (7). 

The association of GIST and adenocarcinoma raises some questions regarding the origin of both cancers. A possibility may be a common progenitor cell capable to differentiate either to mesenchymal and epithelial lineage (9): GISTs represent a mesenchymal neoplasm (2) and pancreatic adenocarcinoma represents an epithelial neoplasm. Moreover, both cancers may be due to the same, at this time unknown, toxin, as suggested in experimental models. For example, in rats, nitrosoguanidine is a tool for production of adenocarcinoma in the stomach, duodenum, jejunum, liver and mesentery (10). However, no data are available on a potentially toxin exposure and development of GIST.

Moreover, it seems to be clinically relevant that the GIST may be asymptomatic in our patients. In fact, twenty-nine GIST in population based study in western Sweden were detected among 14,000 autopsies, suggesting an incidence of 2 per 1,000 population (11); thus, the authors suggested that the true incidence is much higher. 

The data from the literature and our cases suggest that incidental GIST may occur synchronously and metachronously with other cancers more frequently than expected. Thus, the patients with a diagnosis of pancreatic adenocarcinoma may have undergone a strict follow up for GIST.
